# Systemic drug-related intertriginous and flexural exanthema-like eruption after Oxford-AstraZeneca COVID-19 vaccine

**DOI:** 10.1186/s12948-022-00179-8

**Published:** 2022-12-12

**Authors:** Danilo Di Bona, Andrea Miniello, Eustachio Nettis

**Affiliations:** 1 Department of Emergency and Organ Transplantation, School of Allergology and Clinical Immunology, University of Bari Aldo Moro, Policlinico di Bari, Bari, Italy; 2 Unit of Allergology, Azienda Ospedaliero Universitaria Consorziale Policlinico, Bari Piazza Giulio Cesare, 11., 70124 Bari, Italy

## Abstract

Systemic drug-related intertriginous and flexural exanthema (SDRIFE) is an adverse drug reaction which manifests as a symmetrical erythematous rash involving the skin folds after systemic drug exposure. A vast array of possible side effects associated with administration of different anti-SARS-CoV-2 vaccines have been reported in literature since the beginning of the COVID-19 pandemic, but only few times SDRIFE-like eruptions have been described in this context. We discuss here a case of SDRIFE-like eruption following the second dose of Oxford-Astrazeneca Vaxzevria vaccine.

**To the editor,**

A 67-year-old female patient visited our department for an exanthem which occurred few days after receiving the second dose of Vaxzevria (ChAdOx1 nCoV-19; Oxford-AstraZeneca) vaccine.

After the first dose of the vaccine, administered on May 10th, 2021, the patient complained of fever and fatigue for few days. These symptoms showed up again after the second dose which was administered on July 19; 5 days after, she also reported a sharply demarcated pruritic erythematous rash in the inguinal region bilaterally [Fig. 1] that subsequently involved the gluteal fold, the thighs, the lower abdomen [Fig. 2], the inframammary fold and the axillary fold. There was no history of any other constitutional symptom nor any mucosal involvement.

The patient started a treatment with cetirizine without clinical benefit. She was also prescribed topical fluconazole due to suspicion of intertriginous fungal infection by her general practitioner with no improvement. After a few days, the patient consulted a dermatologist who suggested a treatment with topical methylprednisolone acetonate and emollient creams for 20 days, resulting in complete remission. No systemic symptoms were reported throughout the skin rush.

The patient is affected by hypertension, asthma, rhinitis, and contact allergy to nickel and fragrance mix. She didn’t take any drug in the days preceding the appearance of the skin lesions except for her daily oral anti-hypertensive medication (i.e., nebivolol/hydrochlorothiazide), which was never stopped.

Laboratory tests, performed in September, including inflammatory markers and complete blood count were within the normal range. Skin tests (prick and intradermal tests [ID]) with triamcinolone acetonide containing Polisorbate 80 at the concentrations of 40 mg/mL, and 0.4 mg/mL, 4.0 mg/mL, 40 mg/mL, respectively, were negative. The ID tests were read at 15 min and at 96 h. The patient refused skin patch testing and lymphocyte transformation test (LTT), which could have possibly strengthened the suspicion of delayed drug reaction [1], and denied her consent for histological examination.

Based on the patient’s clinical history, the lack of systemic symptoms, of medications taken before the development of the skin lesions, and of response to the anti-fungal agent fluconazole suggested a possible diagnosis (by exclusion) of systemic drug-related intertriginous and flexural exanthema (SDRIFE)-like eruption induced by the COVID-19 vaccine. We applied the Naranjo algorithm for estimation of the probability of adverse drug reaction (ADR), and our case scored 5 points, which is indicative of “probable ADR”. [2]

Cutaneous events associated with COVID-19 vaccination may manifest themselves in many different clinical pictures, but they’re mostly self-limited and easily manageable with topical or oral steroids [3–5]. SDRIFE is a relatively uncommon cutaneous adverse drug reaction mediated by a type IV/delayed hypersensitivity mechanism with only a limited number of cases reported in literature. It is most commonly triggered by antibiotics (especially beta-lactams) [6], but it also been described following the administration of iodinated radiocontrast media and different drugs including antiasthma treatments (aminophylline, terbutaline), allopurinol and monoclonal antibodies (infliximab, golimumab) [7–11]. To date, since the start of the global vaccination campaign, nine cases of SDRIFE-like eruption following COVID-19 vaccines [Table 1] have also been reported. [4, 12–17]. Most of these cases occurred after the second dose, with the exception of the case reported by Manaa et al., which occurred after the third (booster) dose of Pfizer–BioNTech vaccine, and the ones reported by Orenay et al. and Bellinato et al., who did not specify how many injections were administered before the onset of symptoms. The case reported here lends further support to the possible causal relationship between COVID-19 vaccine and SDRIFE.

**Fig. 1:**
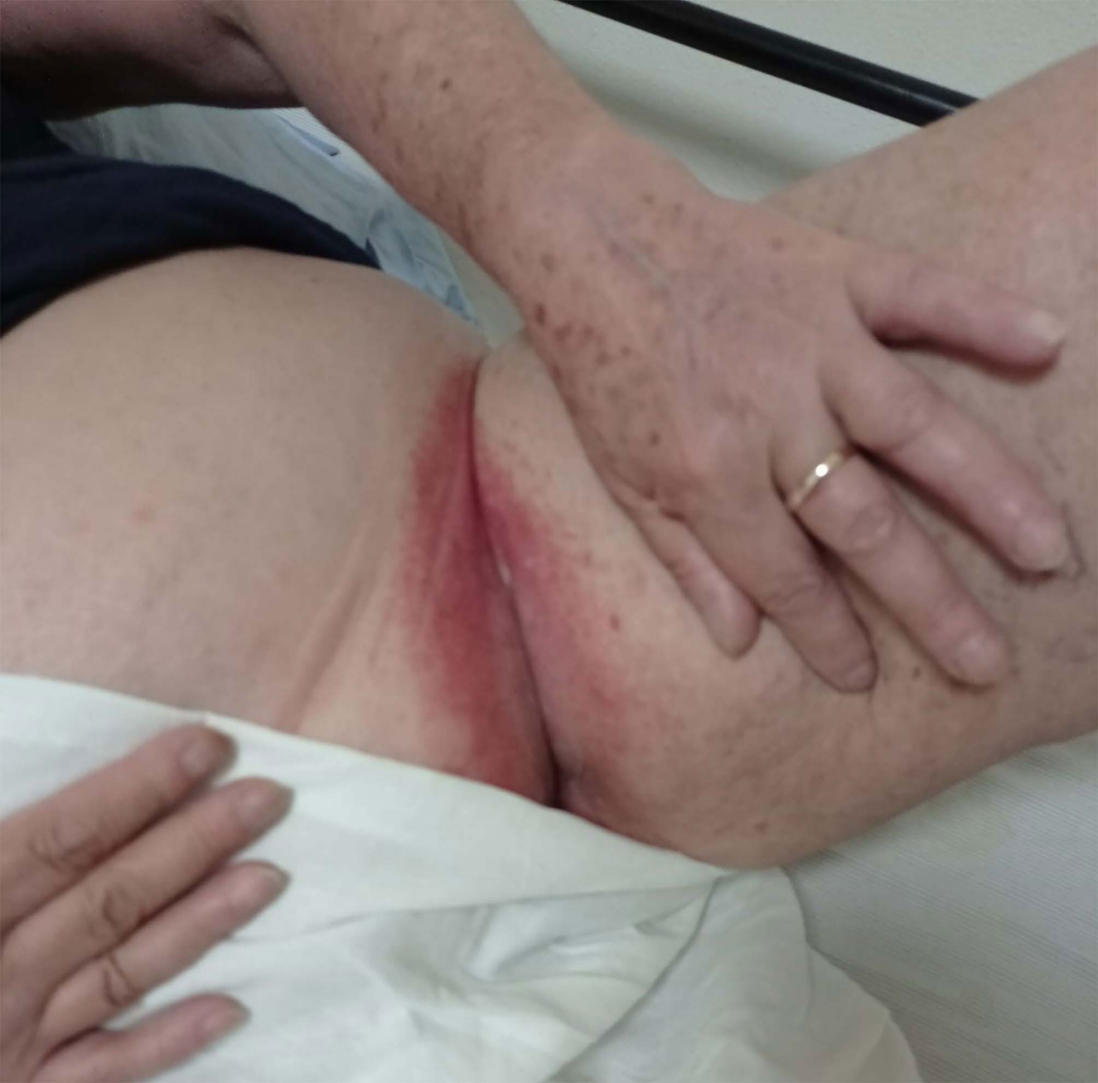
Sharply demarcated erythema on the inguinal region, 12 days after Vaxzevria vaccine second dose

**Fig. 2:**
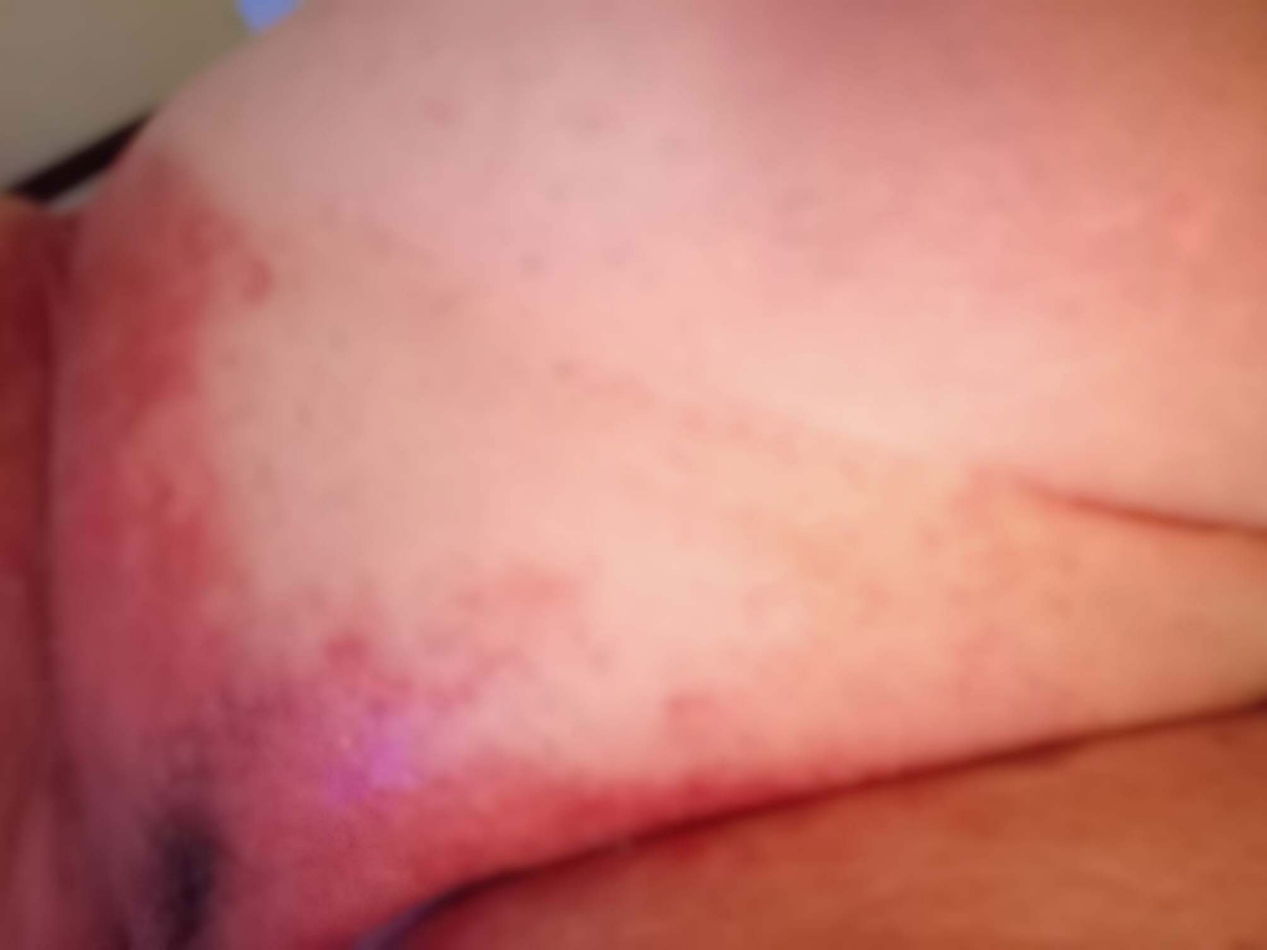
Confluent erythematous papules merging on the lower abdominal region, 14 days after Vaxzevria second dose

**Table 1:** Reported cases of SDRIFE-like eruption secondary following COVID-19 vaccination

Reported case	Vaccine type	Age / sex of patient	Onset after injection	N° of vaccine doses prior to SDRIFE	Treatment
Orenay et al. (2021)^12^	CoronaVac	87 M	4 days	N/A	Oral prednisolone 40 mg/day (3 weeks including tapering off); topical corticosteroids; oral antihistamines
Lim & Wylie (2021)^13^	Vaxzevria (ChAdOx1 nCoV-19)	61 M	1 day	2	Oral prednisolone 30 mg/day (4 weeks including tapering off); topical corticosteroids and antifungals
Hai et al. (2021)^14^	Comirnaty (BNT162b2)	23 M	6 weeks	2	Topical corticosteroid
		38 F	2 weeks	2	Oral prednisolone 40 mg/day (9 days including tapering off); topical corticosteroid
Bellinato et al. (2021)^4^	Comirnaty (BNT162b2)	65 M	2 weeks	N/A	N/A
Hong et al. (2022)^15^	Vaxzevria (ChAdOx1 nCoV-19)	53 M	7 days	2	Oral prednisolone 30 mg/day (2 weeks including tapering off)
Lahouel et al. (2022)^16^	Comirnaty (BNT162b2)	52 F	5 days	2	None (spontaneous remission after 5 days)
	CoronaVac	57 F	3 days	2	Topical corticosteroids; oral antihistamine
Manaa et al. (2022)^17^	Comirnaty (BNT162b2)	59 M	2 days	3	Oral prednisone 40 mg/day (1 month including tapering off); cyclosporine 2.5 mg/kg/day (2.5 months including tapering off); topical corticosteroids
Our case	Vaxzevria (ChAdOx1 nCoV-19)	67 F	5 days	2	Topical corticosteroids (20 days)

## Data Availability

Not applicable.

## References

[1] Tan SC, Tan JWL (2011). Symmetrical drug-related intertriginous and flexural exanthema. Curr Opin Allergy Clin Immunol.

[2] Naranjo CA, Busto U, Sellers EM, Sandor P, Ruiz I, Roberts EA (1981). A method for estimating the probability of adverse drug reactions. Clin Pharmacol Ther.

[3] Gambichler T, Boms S, Susok L, Dickel H, Finis C, Abu Rached N, et al. Cutaneous findings following COVID-19 vaccination: review of world literature and own experience. *J Eur Acad Dermatology Venereol* 2021; published online Feb 1. DOI:10.1111/JDV.17744. 10.1111/jdv.17744 PMC8656409 34661927

[4] Bellinato F, Maurelli M, Gisondi P, Girolomoni G (2021). Cutaneous Adverse Reactions Associated with SARS-CoV-2 Vaccines. J Clin Med.

[5] Sun Q, Fathy R, McMahon DE, Freeman EE (2021). COVID-19 Vaccines and the Skin: The Landscape of Cutaneous Vaccine Reactions Worldwide. Dermatol Clin.

[6] Nespoulous L, Matei I, Charissoux A, Bédane C, Assikar S (2018). Symmetrical drug-related intertriginous and flexural exanthema (SDRIFE) associated with pristinamycin, secnidazole, and nefopam, with a review of the literature. Contact Dermat.

[7] Huynh T, Hughey LC, McKay K, Carney C, Sami N (2015). Systemic drug-related intertriginous and flexural exanthema from radio contrast media: A series of 3 cases. JAAD Case Reports.

[8] Winnicki M, Shear NH (2012). A Systematic Approach to Systemic Contact Dermatitis and Symmetric Drug-Related Intertriginous and Flexural Exanthema (SDRIFE). Am J Clin Dermatology.

[9] Elmariah SB, Cheung W, Wang N, Kamino H, Pomeranz MK. Systemic drug-related intertriginous and flexural exanthema (SDRIFE). Dermatol Online J 2009; 15. DOI:10.5070/D32WS5H2S0. 19891911

[10] Bulur I, Keseroglu HO, Saracoglu ZN, Gonul M (2015). Symmetrical drug-related intertriginous and flexural exanthema (Baboon syndrome) associated with infliximab. J Dermatol Case Rep.

[11] Yang SY, Lan CC, Hu SCS (2017). Symmetrical drug-related intertriginous and flexural exanthema (SDRIFE) induced by golimumab. Int J Dermatol.

[12] Orenay OM, Balta I, Yigit D, Eksioglu M (2021). Systemic drug-related intertriginous and flexural exanthema like eruption after CoronaVac vaccine. J Eur Acad Dermatology Venereol.

[13] Lim PN, Wylie G (2022). Symmetrical drug-related intertriginous and flexural exanthema like eruption associated with COVID‐19 vaccination. Clin Exp Dermatol.

[14] Hai J, Shawa H, Kim-Lim P, Wang JZ, Vy M, Fung MA (2021). Systemic drug-related intertriginous and flexural exanthema induced by the Pfizer-BioNTech COVID-19 vaccine: A report of 2 cases. JAAD Case Reports.

[15] Hong JK, Shin SH, Yoo KH, Li K, Seo SJ (2022). Symmetric drug-related intertriginous and flexural exanthema-like eruption related to coronavirus disease 2019 vaccine. Contact Dermat.

[16] Lahouel I, Ben Salah N, Ben Fadhel N, Chahed F, Ouni N, Belhadjali H, et al. Symmetrical drug-related intertriginous and flexural exanthema-like eruption after COVID-19 vaccine. *J Eur Acad Dermatol Venereol* 2022; published online April. DOI:10.1111/JDV.18108. 10.1111/jdv.18108 PMC9114893 35344627

[17] Manaa A, Ziv M, Krausz J, Dodiuk-Gad RP. A case of symmetrical drug-related intertriginous and flexural exanthema-like eruption associated with Pfizer COVID-19 vaccination. *Dermatol Ther* 2022; published online May 12. DOI:10.1111/DTH.15546. 10.1111/dth.15546 PMC9111842 35485220

